# Endoscopic ultrasound evaluation of portal cavernoma cholangiopathy and endoscopic management of choledochal variceal rupture during ERCP

**DOI:** 10.1055/a-2224-3563

**Published:** 2024-01-09

**Authors:** Ana Paula Samy Tanaka Kotinda, Armelle Poujol-Robert, Audrey Payance, Romain Leenhardt, Marine Camus Duboc, Xavier Dray, Ulriikka Chaput

**Affiliations:** 1 Centre for Digestive Endoscopy, Sorbonne University, Saint-Antoine Hospital, APHP, Paris, France; 2 Gastrointestinal Endoscopy Unit – Gastroenterology Department, Hospital das Clínicas da Faculdade de Medicina da Universidade de São Paulo, São Paulo, Brazil; 3 Department of Hepatology, AP-HP, Hôpital Saint-Antoine, UPMC University, Paris, France; 4 Service d’hépatologie Clinique, Hôpital Beaujon, Clichy, France

Portal cavernoma cholangiopathy (PCC) is characterized by pathological alterations in the biliary system in patients with extrahepatic portal vein obstruction and portal cavernoma 1 . Choledocholithiasis, found in 17% of these patients, is attributed to biliary stasis related to PCC 2 . The biliary alterations in PCC consist of reversible components, caused by extrinsic compression and varices formation, and fixed components, due to fibrosis secondary to ischemic-inflammatory damage from chronic portal vein thrombosis and portal cavernoma 3 4 . This case demonstrates the diagnostic utility of endoscopic ultrasound (EUS) in PCC, the endoscopic treatment of stenosis and choledocholithiasis, and the management of hemobilia resulting from biliary varices rupture ( Video 1 ).

Endoscopic ultrasound evaluation of portal cavernoma cholangiopathy with choledocholithiasis, followed by endoscopic retrograde cholangiopancreatography for choledocholithiasis treatment, complicated by massive bleeding, and managed with placement of a self-expandable metal stent.Video 1

An asymptomatic 60-year-old man with noncirrhotic chronic portal vein thrombosis associated with controlled human immunodeficiency virus infection was referred due to alteration of liver function tests. An abdominal ultrasound showed lithiasis of the common bile duct (CBD). EUS confirmed the presence of collateral vessels ( Fig. 1 ), gallstones in the CBD ( Fig. 2 ), and CBD stenosis consistent with PCC. Endoscopic retrograde cholangiopancreatography (ERCP) was performed for gallstone extraction and stenosis evaluation. Sphincterotomy was uneventful, but balloon sweeping caused significant hemobilia due to CBD varices rupture ( Fig. 3 **a** ), which was successfully managed with placement of a fully covered self-expandable metal stent (FC-SEMS) ( Fig. 3 **b** ).

**Fig. 1:**
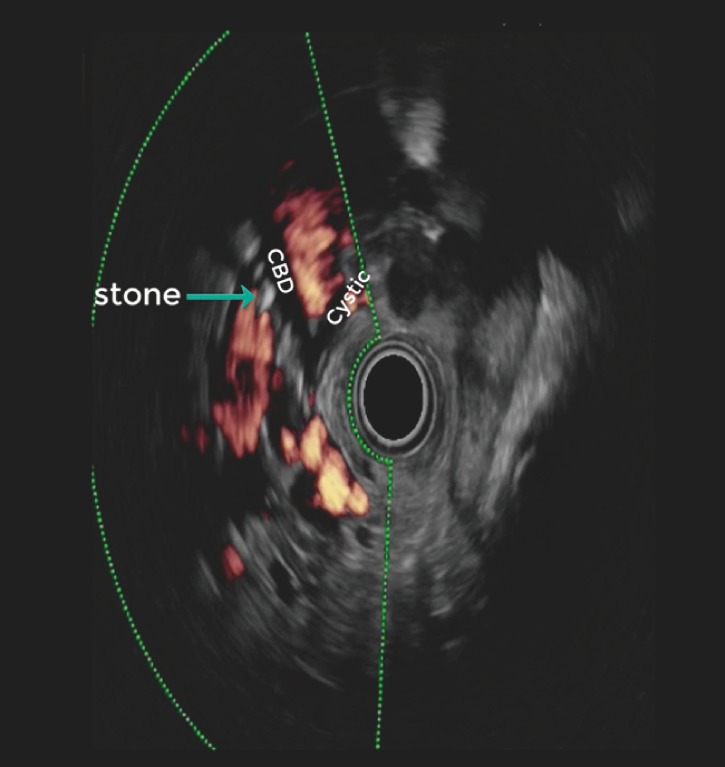
Endoscopic ultrasound image showing the main bile duct and cystic duct, with a gallstone within the common bile duct. Collateral vessels surrounding the biliary tract are also discernible. CBD, common bile duct.

**Fig. 2:**
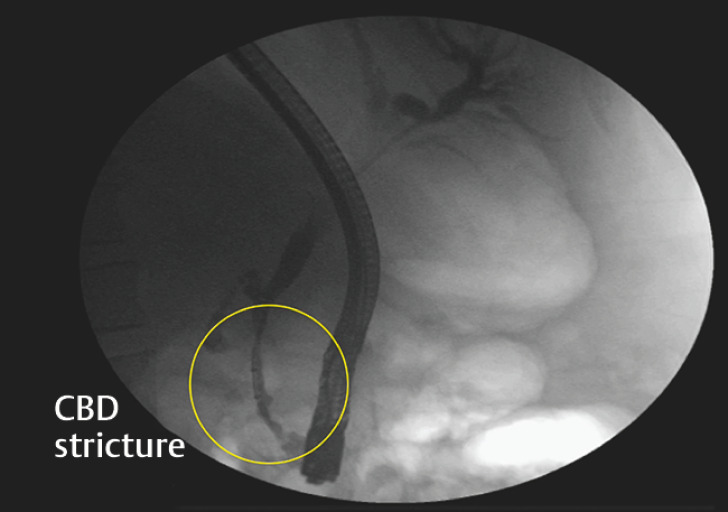
Endoscopic retrograde cholangiopancreatography demonstrated narrowing of the distal common bile duct secondary to portal cavernoma cholangiopathy. CBD, common bile duct.

**Fig. 3:**
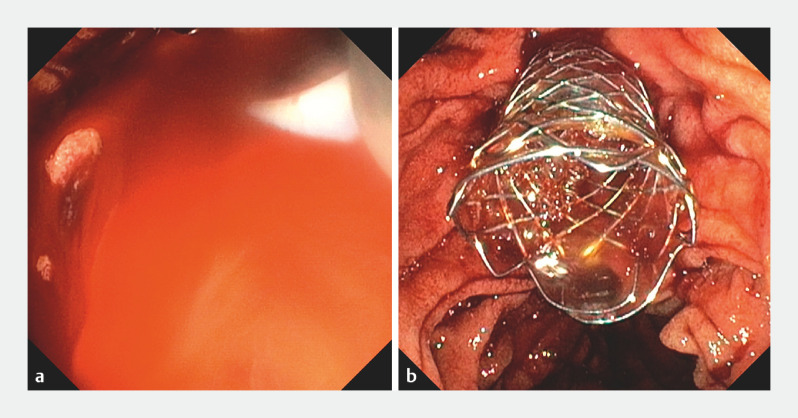
Endoscopic images. **a** Massive bleeding through the biliary tract, caused by rupture of choledochal varices, was observed. **b** Placement of a self-expandable metal stent resulted in good bile drainage and bleeding control.

Portal vein recanalization associated with transjugular intrahepatic portosystemic shunt was performed to complete PCC treatment. Six months after the CBD varices rupture, the previously placed biliary stent was removed during another ERCP, and biliary duct clearance was confirmed, with no active bleeding ( Fig. 4 ). The patient’s condition improved, with preserved liver function and no recurrent bleeding.

**Fig. 4:**
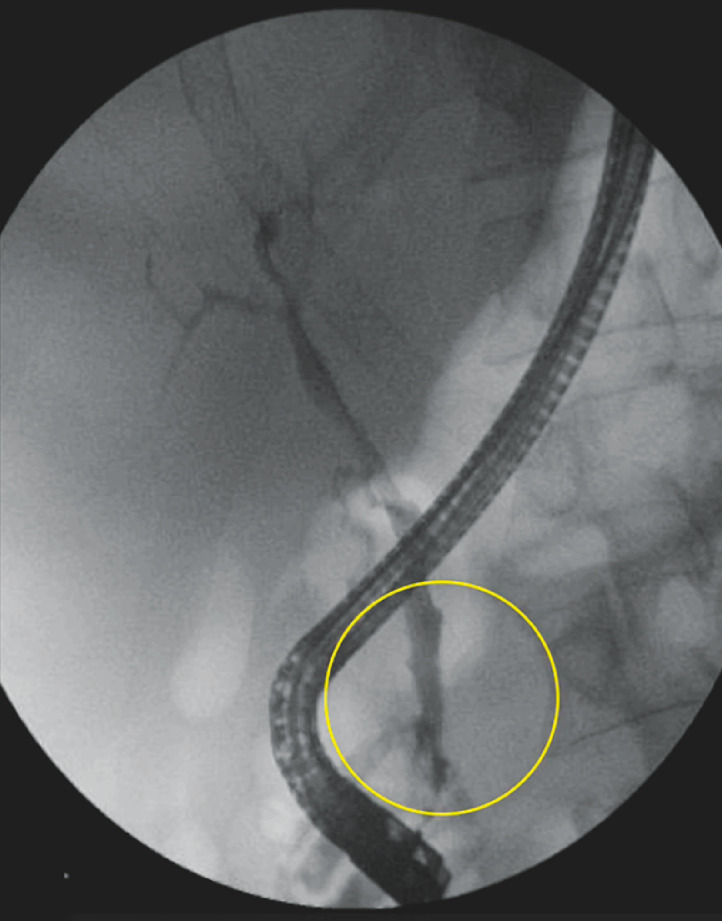
Cholangiography after removal of the self-expandable biliary stent, showing resolution of distal common bile duct stenosis.

This case demonstrates the value of EUS in evaluating PCC and associated choledocholithiasis. It also highlights a rare complication during ERCP and the efficacy of therapeutic interventions employed. Multidisciplinary collaboration among gastroenterologists, hepatologists, and interventional radiologists is crucial for optimizing outcomes in complex PCC cases. These findings contribute to clinical decision making, patient management strategies, and future research in PCC and its associated complications.

Endoscopy_UCTN_Code_CPL_1AK_2AF

## Notes

Endoscopy E-Videos https://eref.thieme.de/e-videos
*E-Videos* is an open access online section of the journal *Endoscopy* , reporting on interesting cases and new techniques in gastroenterological endoscopy. All papers include a high-quality video and are published with a Creative Commons CC-BY license. Endoscopy E-Videos qualify for HINARI discounts and waivers and eligibility is automatically checked during the submission process. We grant 100% waivers to articles whose corresponding authors are based in Group A countries and 50% waivers to those who are based in Group B countries as classified by Research4Life (see: https://www.research4life.org/access/eligibility/ ). This section has its own submission website at https://mc.manuscriptcentral.com/e-videos .
